# The Chemical Characterisation of the Maritime Pine Bark Cultivated in Northern Portugal

**DOI:** 10.3390/plants12233940

**Published:** 2023-11-22

**Authors:** Diana Barros, Élia Fernandes, Meirielly Jesus, Lillian Barros, José Ignacio Alonso-Esteban, Preciosa Pires, Manuela Vaz Velho

**Affiliations:** 1 EDIUS—International PhD School of the USC, University of Santiago de Compostela, 15705 Santiago de Compostela, Spain; 2 CISAS—Center for Research and Development in Agrifood Systems and Sustainability, Instituto Politécnico de Viana do Castelo, Rua Escola Industrial e Comercial de Nun’Álvares, 4900-347 Viana do Castelo, Portugal; eliaf@estg.ipvc.pt (É.F.); meiriellyjesus@ipvc.pt (M.J.); ppires@estg.ipvc.pt (P.P.); mvazvelho@estg.ipvc.pt (M.V.V.); 3 ESTG-IPVC—Escola Superior de Tecnologia e Gestão, Instituto Politécnico de Viana do Castelo, Avenida Atlântico, 644, 4900-348 Viana do Castelo, Portugal; 4 Centro de Investigação de Montanha (CIMO), Instituto Politécnico de Bragança, Campus de Santa Apolónia, 5300-253 Bragança, Portugal; lillian@ipb.pt (L.B.); jignacio.alonso@uah.es (J.I.A.-E.); 5 Laboratório Associado para a Sustentabilidade e Tecnologia em Regiões de Montanha (SusTEC), Instituto Politécnico de Bragança, Campus de Santa Apolónia, 5300-253 Bragança, Portugal; 6 Departamento de Nutrición y Ciencia de los Alimentos, Facultad de Farmacia, Universidad Complutense de Madrid, Plaza Ramón y Cajal, s/n, 28040 Madrid, Spain; 7 Departamento de Ciencias Biomédicas, Facultad de Farmacia, Universidad de Alcalá, Carretera Madrid-Barcelona, Km 33.600, 28805 Madrid, Spain

**Keywords:** bark, *Pinus pinaster* Aiton subsp. *atlantica*, extractives, holocellulose, cellulose, FTIR

## Abstract

Maritime pine, scientifically known as *Pinus pinaster*, holds a vital role in Iberian Peninsula forests, primarily as a source of wood for panels, paper, and cellulose production. Recently, there has been a growing interest in utilising agroforestry by-products to yield valuable chemicals for applications in various sectors, including the food, pharmaceutical, and bioenergy industries. This study aimed to assess the value of the primary by-product of *Pinus pinaster* from the Minho region of northwestern Portugal, i.e., the bark. The research extensively examined the bark’s chemical and thermal characteristics, including ash content, extractives, lignin, cellulose, hemicellulose, fatty acids, and mineral composition. Additionally, various analytical techniques like FTIR, SEM, DSC, DTG, and XRD were used to observe chemical structure differences. The results reveal that the *Pinus pinaster* bark primarily consists of lignin (51.15%) and holocellulose (46.09%), with extractives mainly soluble in toluene–ethanol, followed by water, and a small amount of them are soluble in ethanol. The bark contained around 0.44% ash, and heavy metals such as Cd and Pb were not found. During degradation, *Pinus pinaster* experienced a 10% mass loss at 140 °C. In terms of crystallinity, holocellulose and cellulose showed similar percentages at approximately 25.5%, while α-cellulose displayed the highest crystallinity index at 41%.

## 1. Introduction

The forest industry is an important productive sector based on renewable natural resources. Wood and tree bark contain lignocellulosic materials—cellulose, hemicelluloses, and lignin—as well as extractives [1,2]. Wood, as the primary forest resource, finds extensive application in various sectors such as furniture, construction, and papermaking, and is also used as a renewable energy source [3]. The sawmill industry generates substantial quantities of the bark as a by-product during wood production [4,5]. One of the most available by-products or residues derived in wood-based industries is the bark because it is removed from the tree before processing it (de-barking). Considering the fact that the bark comprises about 9–15% of the total volume of the tree, the amount of waste generated is high [5,6]. Over time, the epidermis gradually accumulates a layer of dead cells, which is commonly known as “bark”. Depending on the species, this layer can weigh between 5% and 28% of the tree’s total weight and acts as a protective structure for trees regarding the environment they are living in, with hazards such as fire, insect pests, and fungi [7,8]. However, due to its woody and outer nature, the bark is typically only utilised for chemical extraction and low-value applications (such as compost amendment) or altogether discarded [7,9]. The defence mechanisms of pine trees (*Pinus* spp.) consist of both mechanical and chemical mechanisms that can be present either inherently or triggered upon challenge. These mechanisms involve a variety of specialised chemicals, including terpenoids found in oleoresin and phenolics, as well as structural features such as thick bark, large resin ducts, and specialised phloem parenchyma cells [10]. The bark contains unique compounds that are not present or vary from those present in the wood [1,11]. The biomass recalcitrance is composed of these components, forming a structure analogous to “reinforced concrete” at various scales [12]. In this analogy, cellulose fibres serve as reinforcement bars, hemicelluloses function as the wire mesh or cables, and lignin acts as the concrete component [13]. The utilisation of lignocellulosic biomass and its primary constituents, namely cellulose, hemicellulose, and lignin, presents a highly attractive opportunity for a multitude of applications. These applications can be found in various areas, including biofuels, cosmetics, detergents, adhesives, biomedical and pharmaceutical devices, flocculants, and food additives [2,14]. These recycled products have low economic value, despite several bioactive compounds that have been identified in bark [15]. Therefore, there exists a great potential for the forest industry to convert and take advantage of these by-products, transforming them into high-value products. 

It is clear that the valorisation of tree bark from the major European forests will lead to a more efficient use of resources, with environmental and economic benefits. The potential availability of pine bark is significant; in Europe alone, it is estimated that ca. 38 Mm3 of bark is available from harvested trees [16]. 

The chemical characterisation of bark, which is essential in assessing its potential uses, has not received as much attention as that of wood. Bark differs from wood in that it has a high concentration of extractives, such as compounds soluble in organic solvents and water, in addition to a significant amount of polyphenolics and inorganic constituents known as ash [17,18]. Analysing the chemical composition of the bark is more complex than that of wood, and the standard methods used for wood analysis may not be suitable for bark due to cross-interference between its components, such as polyphenols and suberin, in the determination of lignin and holocellulose content. Additionally, the structural and anatomical complexity of bark compared to wood makes sampling, characterisation, and processing more difficult.

*Pinus* spp. is an extensive group of conifers from the *Pinaceae* family, mainly distributed around the Northern Hemisphere. Within the more than 100 species of the pine family, *Pinus pinaster*, or maritime pine, is the most commonly used for nutritional and medicinal purposes. Maritime pine (*Pinus pinaster* subsp. *atlantica*) is a conifer native to Mediterranean countries such as France, Spain, and Portugal, as well as some North African countries [19]. Pine is an evergreen, coniferous, resinous, extended-living, and mostly monoecious tree [20]. The subspecies of *P. pinaster* differ from each other in cold and salt tolerance, in addition to their content of terpenes and proteins [21].

The highest area of the Portuguese *P. pinaster* forest was recorded in 1995 (978 × 10^3^ ha), and it has been reduced since that time, with an area of 714 × 10^3^ ha recorded in 2010. Despite this, this species represents an important part of the total forest (about 23%), considered the third most important species after eucalyptus (812 × 10^3^ ha, 26%) and cork oak (737 × 10^3^ ha, 23%) [22,23].

Considering the importance of this pine species for forestry and forest sustainability in Portugal, as well as in the rest of the Iberian Peninsula and other parts of Europe, it is essential to explore all potential uses beyond the wood industry. This involves moving up the value chain to utilise all parts of the tree. Such an approach is closely tied to the principles of the circular economy, which is an economic system that prioritises sustainability by maximising resource usage and minimising waste generation [24].

Considering the interest in the valorisation of agroforest by-products, the present work aims to deepen the knowledge regarding the bark’s chemical composition.

## 2. Results and Discussion

### 2.1. Chemical Composition

There is an abundance of information available on wood, whereas knowledge regarding pine bark is comparatively limited, specifically the chemical composition of maritime pine grown in Portugal. The chemical makeup of pine bark differs from that of wood, with extractives considered the predominant group of compounds [25]. Additionally, the composition of the bark is influenced by various factors such as the tree’s age, location, and growing conditions, as noted by Vázquez et al. (1987) [26]. Other authors, namely Fradinho et al. (2002) [27], Santo et al. (2021) [28], and Elmas and Yilgor (2020) [29], studied dry maritime pines and obtained low values for ash, namely 0.80, 0.40, and 0.38% *w*/*w*, respectively, in line with Alonso-Esteban et al. (2022) [30], who reported an ash content of less than 1% in pine bark. In this study (Table 1), the average ash content was 0.44%, in agreement with the values presented in the literature, namely by Santos et al. (2021) [28]. The lipid fraction, or fat, and the protein content represented 0.98% and 1.94% of the total pine bark composition, respectively, with the value obtained for the fat of the same order of magnitude as that presented by Santos et al. (2021) [28].

Softwood barks, like the bark of maritime pine, were found to have a higher lignin content than the majority of hardwood barks. Additionally, they demonstrated a lower content of acid-soluble lignin, which aligns with the findings observed in wood [34,35]. 

According to the results (Table 1), the primary component of the bark’s overall composition is lignin, which accounts for 51.15% (comprising 48.31% Klason lignin and 2.85% lignin soluble in acid). The high amount of Klason lignin obtained for the maritime pine bark is similar to that reported by Santos et al. (2021) [28], with a Klason lignin percentage of 46%, as well as that presented by Ferreira-Santos et al. (2020) [32], who found lignin to be the predominant component in the total composition of the pine bark, with 41.65% (41.05% Klason lignin and 0.60% acid-soluble lignin). The cellulose content was 23.21%, a higher value than the 17.39% obtained by Ferreira-Santos et al. (2020) [32], although the α-cellulose content of 22.88% was similar to the 23.2% reported by Santos et al. (2021) [28]. The amount of hemicellulose (23.21%) was higher than the levels obtained by other authors, namely Ferreira-Santos et al. (2020) [32] and Santos et al. (2021) [28], with 12.31% and 16.1%, respectively. 

Total extractives represented 9.75% of the sample (*w*/*w*), most of which were soluble in the toluene–ethanol (T:E) mixture (5.61%). Despite being lower, the value of the total extractives obtained was of the same order of magnitude as those presented by Nunes et al. (1996) [31] and Santos et al. (2021) [28], who reported 11.4% and 11.97%, respectively. As in this study, ethanol (E) was used in the first extraction, and the comparison of the extractives was carried out considering the sum of the lipophilic and ethanolic ((L) + (E)) extractives and the hydrophilic (H) ones. This extractive combination is acceptable, as the toluene–ethanol mixture and ethanol are used to remove more hydrophobic extractives (containing lipophilic (L) extractives), such as waxes, fats, resins, and wood gums [36]. When the value found in the combination of extractives of toluene–ethanol and ethanol (7.45%) was compared with the combination of lipophilic and ethanol extractives in the literature, the same tendency was found for total extractives. However, the obtained value for this study was the lowest, with the closest value (8.8%) reported by Nunes et al. (1996) [31]. Despite the differences found for the values in the literature, when the percentage of this extractive fraction in the total extractives was calculated, it was verified that the values were relatively close to the value of this study (76.4%), that is, within an interval of 77.2% [31] to 8.5% [28]. These extractives possibly contained resin acids, such as pimaric and abietic acids, fatty acids, and some other phenolic compounds [37]. Vangeel et al. (2023) [38] also reported that lipophilic extracts contained predominantly resinous acids in the softwood bark, with triterpenoids constituting an important class of lipophilic extracts in the bark. On the other hand, extraction with water allows for the removal of hydrophilic compounds such as tannins, gums, sugars, starches, and pigments. The value found for hydrophilic extractives (2.54%) was very similar to that reported by Nunes et al. (1996) [31]. In the composition of hydrophilic water extractives, there was the possibility of the presence of carbohydrates (that is, arabinose and ribose derivatives) and levulinic acid, as reported by Fernandes (2020) [37]. Thus, the ethanolic extracts obtained from the maritime pine bark probably have phenolic compounds, and the aqueous extracts likely have some short-chain sugars. The ratio of lipophilic and ethanol extracts to hydrophilic extracts was 2.9, in agreement with other authors who obtained values in the range of 3.4 to 4.7. These results seem to indicate that the amount of hydrophilic extractives is much lower than the other extractives. 

The complete extraction used a combination of different solvent polarities; thus, toluene–ethanol removed nonpolar groups more significantly, while water removed polar structures and ethanol groups with similar chemical solubility [39]. In other words, the combination of sequential extractions using solvents with a wide range of polarities seems to be advantageous for the extraction of both lipophilic and hydrophilic fractions.

The lignocellulosic material cellulose is normally obtained through bleaching and alkaline treatment processes. In this study, two processes were carried out to obtain cellulose, an acid hydrolysis using a CH_3_COOH-HNO_3_ mixture to obtain crude cellulose and an alkaline treatment using NaHO to obtain α-cellulose from holocellulose (Table 2). The colour observed for the crude cellulose was brownish-yellow. In the alkaline hydrolysis process, the brownish-yellow colour of holocellulose changed to the yellowish-white colour of α-cellulose, with this colour change possibly resulting from the removal of noncellulosic materials such as residual lignin and hemicellulose. The results showed variations in the brightness and whiteness index (WI) with the type of hydrolysis performed. The results pointed to lower whiteness index values for holocellulose, followed by crude cellulose, with α-cellulose having the highest values for that parameter. The use of different hydrolysis agents showed different whiteness indexes for the cellulose obtained, with the alkaline hydrolysis process resulting in a higher degree of whiteness (higher L* value; brightness). It should be noted, however, that this alkaline hydrolysis was performed on a holocellulose sample that had already undergone a lignin removal process. Thus, the degree of whiteness seems to have been influenced by the content of noncellulosic materials such as lignin and hemicellulose [40], that is, the lower the lignin content in the cellulose, the greater the degree of whiteness [41].

### 2.2. Fatty Acid Composition

Thirteen fatty acids were identified in the pine bark lipid extract (Table 3), and among the identified fatty acids, eleven were saturated, one was monounsaturated (MUFA—oleic acid (18:1*n*-9)), and one was polyunsaturated (PUFA—α-linoleic acid (18:2*n*-6)). The chain length of the fatty acids ranged from 12 to 24 carbon atoms. The saturated fatty acid content was much higher than the unsaturated fatty acid content, ca. 11.9-fold. Thus, the fatty acid composition showed mainly saturated fatty acids, representing approximately 92% of the total fatty acid profile, in line with the 90% registered in the study by Sousa et al. (2018) [42]. Indeed, lignoceric acid (24:0) and behenic acid (22:0) were the major fatty acids, both classified as very long-chain saturated fatty acids, with 34.48% and 32.47% of the total fatty acids, respectively. These were followed by palmitic acid (16:0) and arachidic acid (20:0), with 9.84% and 5.91%, respectively. In other words, only four fatty acids contributed more than 80% (82.7%) of the total fatty acids found. The saturated fatty acids found in higher amounts coincided with those observed in the studies by Kurtça and Tumen (2020) [43], Simões et al. (2021) [10], and Sousa et al. (2018) [42], however, their relative abundance was different, being only similar to those found by Sousa et al. (2018) [42]. Oleic acid (18:1*n*-9) was the unsaturated fatty acid with the highest concentration, representing 5.43% of the total fatty acids. In the studies by Sousa et al. (2018) [42], Kurtça and Tumen (2020) [43], and Simões et al. (2021) [10], oleic acid was also the most representative of the unsaturated fatty acids, although in a higher percentage than that shown in this study. The fatty acids detected in this study were similar to those detected in other studies; however, the percentage composition of the fatty acid profile differed from study to study, with the study by Sousa et al. (2018) [42] presenting more similar values. The results of the comparison showed that, although the main components were similar in general, the fatty acid content was different. The differences shown in the fatty acid profile of this study and those reported by other authors may be due to the time of year in which the pine bark was collected, as verified by Kurtca and Tumen (2020) [43].

### 2.3. Mineral Analysis

As the SEM-EDS (scanning electron microscopy–energy-dispersive spectroscopy) method is a semiquantitative method to determine elemental concentrations, to verify the acceptability of its quantification, the P concentration was also determined using the MAE-Vis (molecular absorption spectrophotometry) method, and the values were compared. The values obtained were 57.9 and 61.8 mg/kg dry matter using the MAE-Vis and SEM-EDS methods, respectively (Table 4). Considering the value obtained for P using MAE-Vis as the reference value, the relative error of the SEM-EDS determination of that element was 6.7%. Since the relative error of the SEM-EDS determination was <10%, the values obtained with that method were considered acceptable.

In the inorganic fraction of pine bark (ash), 18 elements were determined, and 16 were quantified, with their concentrations in descending order as follows: O, Ca, Al, Mg, C (inorganic), K, Si, Na, S, Fe, P, Mn, Zn, Cu, Cr, and Ni (Table 4). The elements with the highest concentration were O, Ca, Al, Mg, and C (inorganic), in the range between 2088 and 199 mg/kg dry matter. Those elements were followed by a set of elements consisting of K, Si, and Na, with concentrations of 134, 125, and 93 mg/kg dry matter, respectively. Each of the aforementioned elements presented a global contribution of 4167 mg/kg of dry matter (0.42 g/100 dry matter), that is, 0.42% of the total dry matter, which represents 95% of the inorganic fraction (ash) of pine bark. 

The elements S, Fe, P, and Mn showed concentrations in the range of 67 to 34 mg/kg dry matter. Fe and Mn are important elements, as plant photosynthesis, redox processes, nitrogen metabolism, and nucleic acid metabolism in conifers are influenced by these elements. The excessive presence of manganese hinders the absorption and movement of iron in plant tissues. That antagonistic relationship can also be observed, where a high iron concentration decreases the absorption and effectiveness of other metals [44,45]. It should be noted that sulphur is an important element both for the structure of proteins [46] and for the functioning of enzymes [47], in addition to playing roles in maintaining the homeostasis of essential micronutrients such as iron (Fe), copper (Cu), zinc (Zn), and manganese (Mn) [48]. Furthermore, this element plays an important role in plant defence against stress and pests [47].

The elements Cu, Cr, Ni, and Zn showed concentration values lower than 3.2 mg/kg dry matter, and Cd and Pb were below the detection limit. Heavy metals, such as Cd, Pb, Cu, Zn, Cr, Ni, and Hg, are normally present in the environment. Although some of these metals serve as essential micronutrients for plant growth, Cd, Pb, and Hg are exceptions to this rule [45,49]. In the analysed material, the Cu content found was 1.76 ± 0.037 mg/kg; however, previous studies reported higher Cu levels in pine bark, ranging from 2.54 to 4.03 mg/kg [50]. Conifers demonstrate resilience to elevated copper levels in the environment and have the capacity to accumulate substantial quantities of this metal [51]. The Zn content was 3.11 ± 0.050 mg/kg. Compared to other heavy metals, zinc is typically found in higher proportions in the environment [45]. In this study, the Ni content was 0.33 mg/kg. However, Poikolainen (1997) [50] reported a nickel content ranging from 0.79 to 2.43 mg/kg in pine bark. Nickel is classified as a micronutrient, particularly for urease and hydrogenases [45]. Coniferous trees demonstrate resilience to high environmental nickel concentrations, with pine trees belonging to the group of Ni hyperaccumulators [51]. The Cr concentration was 0.429 ± 0.0077, in agreement with the values obtained by Kirchner et al. (2008) [51], that is, chromium contents below 1 mg/kg in pine wood (*Pinus jeffreyi*). Likewise, Liu et al. (2018) [52] and Poikolainen (1997) [50] reported chromium of 0.5 mg/kg and 0.12–7.31 mg/kg contents in the pine bark, respectively. The elements Cu, Zn, Ni, and Cr are classified as micronutrients and play a crucial role as constituents of numerous plant enzymes [53] and plant growth regulators [45]. In the tested samples, the concentrations of Cd and Pb were below the detection limit (<0.060 mg/kg and 0.75 mg/kg, respectively), which seems to indicate that the tested maritime pine bark was not contaminated with these metals. For comparison, Kirchner et al. (2008) [51] found cadmium levels of 0.02–0.17 mg/kg in pine wood, and Poikolainen (1997) [50] found levels of 0.10–0.23 mg/kg in the bark of pine trees growing in areas with different degrees of contamination. The results obtained for this set of heavy metals generally showed lower values than those reported in the literature, which seems to indicate that the pine bark tested in this study did not suffer major contamination from them. The total mass of elements determined was 4397 mg/kg of dry matter, representing 99.94% of the ash content, which seems to indicate that the quantification of elements in the sample inorganic fraction was almost complete.

The heavy metal concentration is influenced by the age and specific anatomical part of the plant, as well as by environmental pollution, mainly dust levels [49]. Thus, heavy metals in wood waste may originate from natural uptake by plant roots or from dust settling on tree surfaces such as bark, needles, and cones [49,54,55,56,57]. Consequently, the surfaces of coniferous trees tend to exhibit higher levels of atmospheric dust-derived metals [55,58]. It is important to consider the presence of soil particles and other unwanted fine materials in the bark, especially when it comes to pine bark, as it has a macroscopic structure that is more likely to capture foreign particles, which can be released during the milling process.

### 2.4. Fourier Transform Infrared (FTIR) Analysis

**Figure 1:**
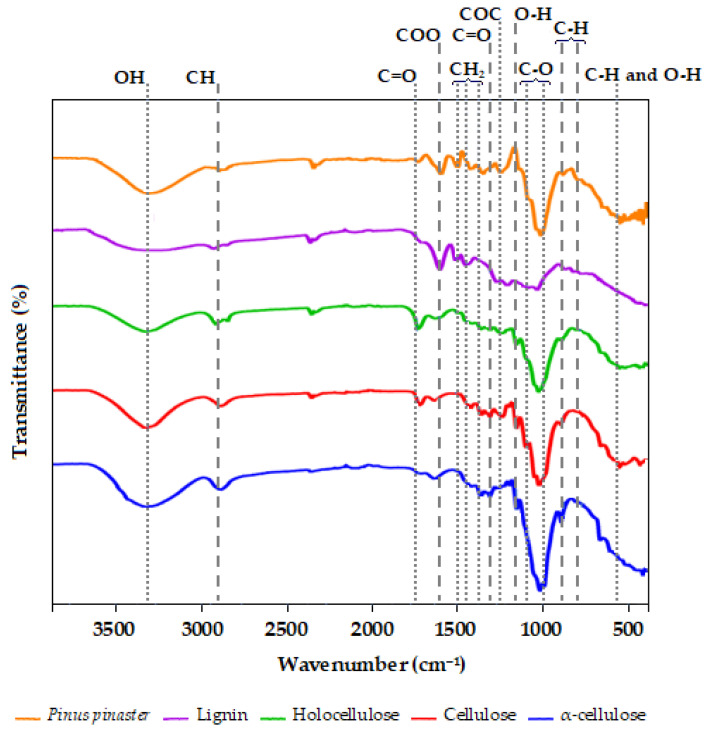
Characterisation of *Pinus pinaster* and their fractions: lignin, holocellulose, cellulose, and α-cellulose.

The FTIR spectra of *Pinus pinaster* and its fractions ranging from 4000 to 600 cm^−1^ are illustrated in Figure 1. The spectra depict the intricate nature of this biomass, resulting in a diverse array of characteristic peaks corresponding to its constituents. As stated by Wang et al. (2020) [59], the peaks at 1425, 1169, and 890 cm^−1^ observed in all samples except for lignin are attributed to typical cellulose structures. The transmittance between 3316 and 3339 cm^−1^ in the samples corresponds to the hydroxyl bond of the water molecule [60,61]. Additionally, it is associated with OH stretching, revealing hydrogen bonding and intramolecular interactions within the cellulose component of the wood [62]. The peaks in the range of 2945–2878 cm^−1^ could be ascribed to symmetric and asymmetric CH-stretching vibrations originating from the methyl (-CH_3_) and methylene (>CH_2_) functional groups [63]. The peaks within the 738–1614 cm^−1^ range were assigned to the stretching vibrations of C=O in carboxyl, and certain segments of the acetyl group were found in hemicellulose, as well as vibrations related to the aromatic skeleton. The symmetric stretching bands of the carboxyl group within the 1436–1319 cm^−1^ range were associated with CH deformation (methyl and methylene), CH_2_ bending vibrations, and CH_3_ stretching. Additionally, this transmittance could be linked to the crystalline structure of cellulose and its derivatives [64,65]. The peaks within the range of 1261–1204 cm^−1^ were ascribed to COC vibrations in both cellulose and hemicellulose. For the pine bark biomass, peaks at 1257 cm^−1^ were detected, which can be attributed to C–O stretching in lignin and the C–O linkage in the aromatic methoxyl groups, particularly guaiacil, and this may have a direct association with softwoods [29]. These characteristic peaks are indicative of the presence of cellulose, hemicellulose, and lignin. The prominent bands falling within the range of 1003–1029 cm^−1^ originated from the CO-stretching vibration in cellulose, hemicelluloses, and lignin. Slight variations in the peak heights were noted between holocellulose and pine bark biomass samples, a finding that was consistent with observations by Elmas and Yilgor (2020) [29].

For the lignin sample, the spectra exhibited peaks at 1601, 1511, and 1452 cm^−1^ corresponding to vibrations of the aromatic ring in the phenylpropane skeleton. Furthermore, the broadband at 3337 cm^−1^ was directly linked to both aromatic and aliphatic OH groups, while the peaks observed at 2945 and 1457 cm^−1^ corresponded to CH vibration in the CH_2_ groups. The bands at 1262, 875, and 798 cm^−1^ were associated with guaiacil (G) type units, which are abundant in softwoods like *Pinus* [29,66]. A lesser-intensity peak at 1212 cm^−1^ was likely a result of the OH-bending vibration. These findings align with those reported in the literature for lignin derived from pine and other similar softwoods [59,67,68,69].

### 2.5. Scanning Electron Microscopy (SEM) Analysis

**Figure 2:**
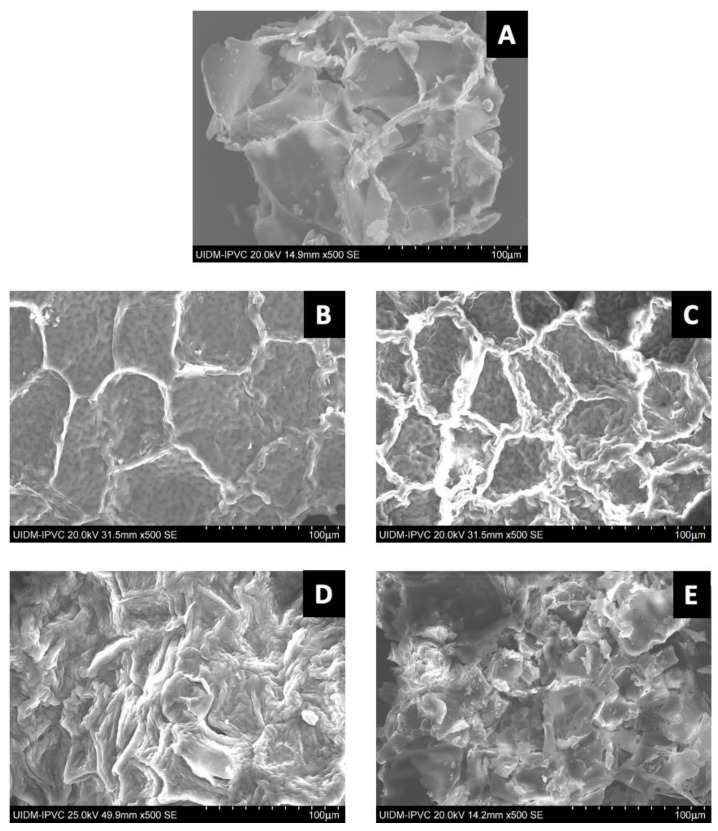
Displays micrographs at 500× magnification of maritime pine bark biomass (**A**) along with its isolated components, including holocellulose (**B**), cellulose (**C**), α-cellulose (**D**), and lignin (**E**).

The morphology of pine bark biomass (A) and its fractions (holocellulose (B), cellulose (C), α-cellulose (D), and lignin (E)) were examined through scanning electron microscopy (SEM), as illustrated in Figure 2. The pine bark sample (Figure 2A) exhibits a compact, smooth, and rigid surface structure attributed to the presence of lignin, which envelops the integral fibrils of hemicellulose and cellulose [63,70]. In Figure 2B, the micrograph of holocellulose reveals a uniform and distinctly defined structure. These characteristics are likely attributed to its composition, primarily consisting of cellulose and all hemicelluloses. Holocellulose was obtained by removing extractives and lignin from the original natural material, resulting in the complete polysaccharide fraction of lignocellulosic biomass [71]. 

Figure 2C seems to indicate that the isolation procedure may have had a significant impact on the morphology of the cellulose, resulting in a rough and irregular surface. This may have occurred due to the fragmentation of the lignocellulosic structure and the removal of the hemicellulose and lignin components. The removal of these elements from the cell wall may have directly affected the organisation that interconnected the cellulose fibrils [72]. These findings appear to be in line with FTIR analysis, which points to the removal of lignin and hemicellulose. Notably, α-cellulose, shown in Figure 2D, exhibits aggregated and elongated fibres. These results are consistent with the literature, as some authors described cellulose and its derivatives as having a fibrous morphology with a rough and irregular appearance [73]. The micrograph of lignin isolated from pine bark and presented in Figure 2E shows a heterogeneous morphology with significant ruggedness, in agreement with findings in the literature, which characterised lignin as having an irregular surface with variations and undulations [74].

### 2.6. Thermal Analysis of Initial Material (Pinus pinaster Cortex)

**Figure 3:**
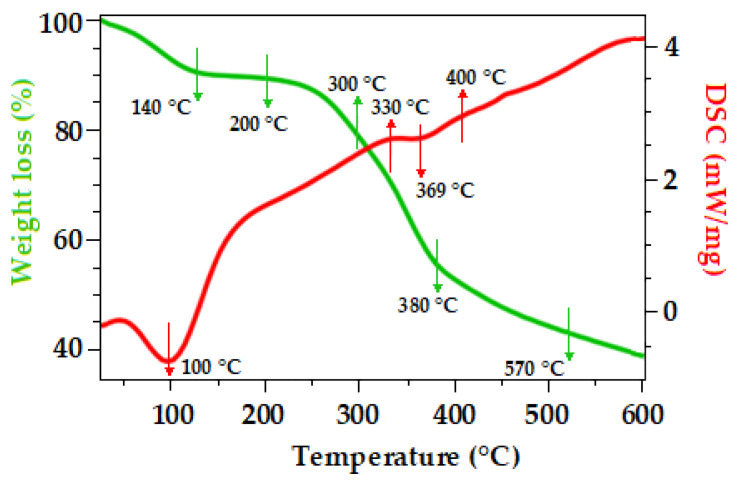
DSC and DTG curves of Pinus pinaster bark.

Thermogravimetric analysis is a cost-effective method used to evaluate the thermal behaviour of lignocellulosic biomass under specific atmospheres and either isothermal or nonisothermal conditions [75]. Gaining insight into the thermal properties of lignocellulosic biomass is essential for grasping the physicochemical alterations that occur during pretreatment [76]. Thermogravimetric analysis is usually depicted through differential thermogravimetry (DTG) curves, which depict mass loss concerning temperature and time. On the other hand, differential scanning calorimetry (DSC) serves as a nonspecific diagnostic instrument employed to capture the entire spectrum of phase transitions occurring during a temperature scan in a single step [77,78]. The thermogravimetric analysis of the Pinus pinaster bark is illustrated in Figure 3. 

In the Pinus pinaster bark, the DSC temperature ramp revealed the initial reaction at approximately 100 °C, primarily associated with the elimination of moisture within the sample. Further analysis indicated two exothermic reactions occurring at 330 °C and 400 °C, corresponding to hemicellulose and lignin, along with an endothermic reaction at 369 °C, representing cellulose. These thermal events showed the interactions among the primary components of this lignocellulosic residue [78,79].

The biomass underwent four distinct stages of weight loss, with the initial stage occurring below 140 °C, the second within the 250–300 °C range, the third at 380 °C, and the fourth at 570 °C, as evident in the DTG curves depicted in Figure 3. During the first stage, a minor weight loss of approximately 10% was observed from room temperature to 140 °C, primarily attributed to the evaporation of water and physically adsorbed moisture [75]. Furthermore, the observed mass loss between 200 and 300 °C may be associated with the decomposition of hemicelluloses and pectins, which are more thermally degradable [80,81]. The decomposition stages that occurred between 300 and 380 °C were attributed to the degradation of cellulose [76]. As pointed out by Yang et al. (2007) [71], in contrast to hemicellulose, cellulose is composed of an unbranched, long polymer of glucose with a highly organised and robust structure, which contributes to its enhanced thermal stability. As for lignin, its decomposition began between 380 and 570 °C, lignin is an aromatic heteropolymer compound composed of phenyl propane units. These units impart increased thermal stability to lignin, necessitating higher temperatures for its degradation [76,82].

### 2.7. X-ray Diffraction (XRD) Analysis

**Figure 4:**
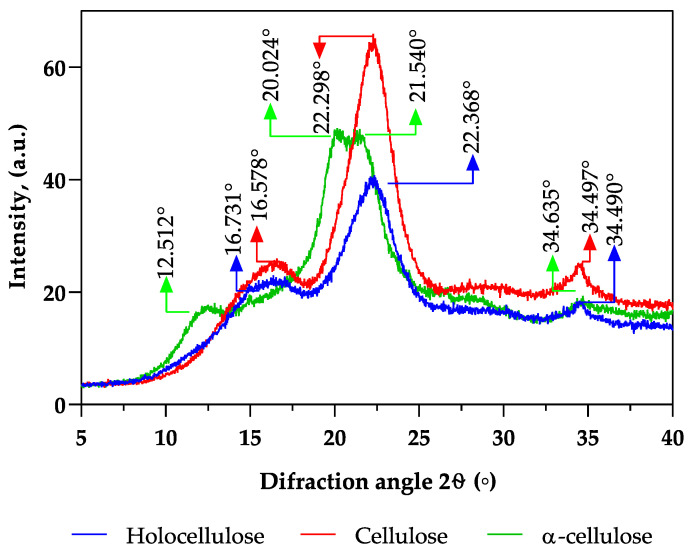
X-ray diffraction patterns of separated components from pine bark, specifically hemicellulose, cellulose, and α-cellulose.

The crystallinity index (CrI) is often used to assess the crystalline fraction of cellulose materials and monitor the changes resulting from various physical–chemical and biological treatments. This parameter is based on distinguishing between a crystalline phase and an amorphous phase of the material. The XRD diffraction patterns of holocellulose and cellulose shown in Figure 4 exhibit three distinct diffraction peaks near the 2θ angles of 16°, 22°, and 34°, revealing that the crystalline structure of the samples was cellulose, as described in previous literature [83]. However, α-cellulose exhibits a broader 2θ peak ranging from 15.0° to 25.0°; these variations may be possibly due to the highly amorphous nature of the synthesised cellulose [73].

Concerning the crystallinity index, both holocellulose and cellulose showed similar percentages, at approximately 25.5%. On the other hand, α-cellulose displayed the highest crystallinity index at 41%. The high crystallinity of the α-cellulose sample seemed to indicate that the depolymerisation of hemicellulose and delignification were successful during the extraction process [84].

## 3. Materials and Methods

### 3.1. Sampling

In the northern region of Portugal, specifically in Valença, the pine bark sourced from *P. pinaster* was gathered in May 2021. These trees were part of an experimental and certified plantation situated in the forest area of the Minho region. It is noteworthy that the collected pine bark originated from trees that were precisely 21 years old. Pine bark samples were collected by making a circular cut in an area of the main trunk of the freshly cut tree, about 1.30 cm above the ground. 

### 3.2. Sample Preparation

The pine bark was washed with distilled water several times to remove dirt, lichens, and resin. The bark was dried at 40 °C for 48 h and subsequently milled and sieved at an amplitude of 0.2 for 1 min (Analysette 3 PRO, Fritsch, Idar-Oberstein, Germany) to select the particles from 200 to 850 µm diameter. Finally, the ground pine bark was kept in sealed bags and stored in a dry and dark place until further analysis. 

### 3.3. Experimental Design

**Figure 5:**
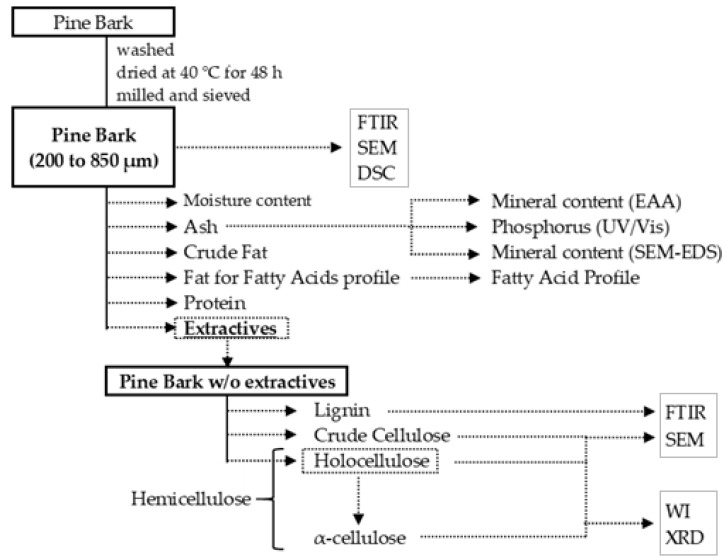
Experimental design of the study.

The experimental design followed to study the composition of pine bark is shown in Figure 5.

### 3.4. Chemical Composition of Pine Bark

The pine bark was analysed for moisture, ash, proteins, and fat contents using the AOAC procedures [85]. The moisture was determined by drying in an oven at 103 °C until constant weight (AOAC 930.04); the ash content was determined via incineration at 550 °C (AOAC 930.05); the crude protein content (N x 6.25) of the samples was estimated using the Kjeldahl method (AOAC 978.04); and the crude fat was determined by extracting a known weight of ground sample with petroleum ether using a Soxhlet apparatus (AOAC 920.39).

The analysis of the chemical composition of the pine bark also included the determination of extractives using the solvents toluene–ethanol (T:E), ethanol (E), and deionised water (H), as well as crude cellulose, α-cellulose, hemicelluloses, and lignin.

*Extractive content*: The samples were extracted successively with toluene–ethanol 95% (2:1 by volume) for 6 h, ethanol 95% for 4 h, and distilled water for 2 h in a Soxhlet apparatus. The extractive content was determined from the extracts’ dry matter at 105 °C and reported as a percentage of the sample dry matter according to the ASTM D 1105-96 method (2001) [36] and Ona et al. (1995) [86]. 

*Lignin*: The acid-soluble and acid-insoluble lignin contents were determined based on a protocol adapted from the method described by Sluiter et al. (2008) [87]. This procedure uses a two-step acid hydrolysis to fractionate the biomass into forms that are more easily quantified. In the first stage of acid hydrolysis, 3 mL of 72% sulphuric acid was added to 300 mg of the sample, and the mixture was homogenised for 1 min. The mixture was placed in a water bath set at 30 ± 3 °C and incubated for 60 ± 5 min, with stirring every 5 to 10 min. The tube was removed from the water bath, and the sample was quantitatively transferred to an autoclave flask. The acid concentration in the flask was diluted to 3.5% by adding distilled water until the mixture weighed 89.21 g. In the second step of acid hydrolysis, the sample was autoclaved for 1 h at 121 °C. The hydrolysate was allowed to cool slowly to near room temperature. Subsequently, the samples were vacuum-filtered through a glass fibre filter conditioned at 575 °C (GF/C, Whatman, Maidstone, England). The filter was washed with 3.5% sulphuric acid without exceeding the volume of 100 mL. The filtrate was transferred to a 100 mL volumetric flask, and the volume was completed with 3.5% sulphuric acid. 

*Acid-soluble lignin*: The absorbance was measured at 240 nm against 3.5% sulphuric acid (Hitachi U 1100 Spectrophotometer, Tokyo, Japan). Absorbance should be in the range of 0.7–1.0.

*Acid-insoluble lignin*: The filter with the residue was washed with at least 50 mL of distilled water, dried at 105 ± 3 °C to a constant weight (>4 h), cooled, and weighed to the nearest 0.1 mg. The filter was placed in a muffle at 575 ± 25 °C for 24 ± 6 h, cooled, and weighed to the nearest 0.1 mg. Acid-insoluble material can also include ash and protein, which must be considered during gravimetric analysis.

*Crude cellulose*: Of the different cellulose determination methods proposed in the literature, the one involving a mixture of acetic acid and nitric acid has the advantage of being chlorine-free, reducing the environmental impact associated with that compound. Furthermore, this methodology has the advantage of being able to achieve a high degree of delignification and removal of noncellulose polysaccharides in a single-step procedure [88]. Based on the method described by Sun et al. (2004) [89], to 0.5 g of pine bark sample, 50 mL of an 80% acetic acid/70% nitric acid mixture (*v*/*v*, 10:1) was added in a reflux flask. The mixture was heated to the boiling point and refluxed for 25 min with periodic stirring. Subsequently, the hot mixture was filtered under vacuum, and the filter was washed with 5 mL of the hot acidic mixture. Sequentially, the filter was washed with hot water until it reached a neutral pH, several times with ethanol (95%), and with ethyl ether. Finally, the solid was dried at 105 ± 3 °C until it reached a constant weight.

*Holocellulose*: In a 250 mL Erlenmeyer flask, 180 mL of 0.2% sodium acetate (pH 3.5), 0.5 mL of acetic acid, and 1.2 g of sodium chlorite were added to 3 g of sample. The mixture was heated in a water bath at 70 °C. After 30 min, 0.5 mL of acetic acid and 1.2 g of sodium chlorite were added. After each successive hour, fresh portions of 0.5 mL of acetic acid and 1.2 g of sodium chlorite were added. The delignification process degrades some of the polysaccharides, and the application of excessive chlorination should be avoided. The addition of 0.5 mL of acetic acid and 1.2 g of sodium chlorite was repeated until the bark sample was completely separated from the lignin. Chlorination usually takes 6 to 8 h, and the sample can be left without adding acetic acid and sodium chlorite in the water bath overnight. At the end of 24 h of reaction, the sample was allowed to cool, the holocellulose was filtered through filter paper in a Buchner funnel, and washed with distilled water until the yellow colour (the holocellulose colour is white) and the chlorine dioxide odour had been removed. The filter was washed with acetone, dried at 105 °C for 24 h, cooled, and weighed. Holocellulose may contain some lignin, which should be considered during gravimetric analysis.

*α-Cellulose*: The α-cellulose was determined following holocellulose hydrolysis. In a 200 mL beaker, 25 mL of 17.5% sodium hydroxide was added to 1 g of the sample (holocellulose) and stirred. After 4 min, the sample was smashed for 5 min. After 16 min, 25 mL of distilled water was added, and the sample was stirred for 1 min. After 16 min, 25 mL of distilled water was added, and the sample was stirred for 1 min. After 5 min, it was filtered under vacuum through a glass filter conditioned at 105 °C. The filter was washed with distilled water until it reached a neutral pH. A 40 mL volume of 10% acetic acid was added and kept for 5 min. The filter was washed again with distilled water until it reached a neutral pH, and it was dried at 105 °C until it reached a constant weight [86].

*Hemicellulose*: Hemicellulose was calculated by subtracting α-cellulose from holocellulose. Data are expressed in weight percentage.

The determination of moisture, ash, crude protein, crude fat, and crude cellulose was carried out in the washed, milled, sieved, and dried pine bark sample (sample preparation), lignin and holocellulose in the sample after removing extractives, and α-cellulose in the holocellulose sample.

*Degree of Whiteness*: The degree of whiteness of holocellulose, cellulose, and α-cellulose was measured using a colourimeter (CR-400, Konica Minolta, Ramsey, NJ, USA). The CIELab colour system value (L*, a*, b*) was used to estimate the degree of whiteness, where L* is lightness, a* is redness, and b* is yellowness. The L* value ranges from 0 to 100 (black to white). The value of +a* (positive) describes a red shift, and −a* (negative) indicates a green shift; the value of +b* (positive) describes a yellow shift, and −b* (negative) indicates a blue shift.

### 3.5. Fatty Acid Composition

Fatty acids were quantified via gas–liquid chromatography with flame ionisation detection (GC–FID)/capillary column as previously described by Pereira et al. (2011) [90]. First, the transesterification of the fatty acids was carried out from the previously extracted fat. For this, 5 mL of methanol–sulphuric acid–toluene 2:1:1 (*v*:*v*) was added, and the reaction occurred for at least 12 h in a bath at 50 °C and 160 rpm; then, 3 mL of deionised water was added, fatty acid methyl esters (FAMEs) were recovered with 3 mL of diethyl ether by shaking in the vortex, and the upper phase was passed through a micro-column of sodium sulphate anhydrous, in order to remove any aqueous residue; the sample was recovered in a vial with Teflon, and before injection, the sample was filtered with 0.2 μm nylon filter. The FAMEs were analysed with a DANI model GC 1000 instrument equipped with a split/splitless injector, a flame ionisation detector (FID), and a Macherey–Nagel column (30 m × 0.32 mm ID × 0.25 μm df), according to Barros et al. (2010) [91]. FAME identification was carried out by comparing the relative retention times with standards. The results were recorded and processed using CSW 1.7 software (DataApex 1.7) and expressed in relative percentage of each fatty acid.

### 3.6. Mineral Analysis

The mineral analysis was carried out on the ash of the sample. To determine the concentration of cadmium (Cd), chromium (Cr), copper (Cu), lead (Pb), nickel (Ni), phosphorus (P), and zinc (Zn), the ashes were hot-dissolved with nitric acid and the resulting solution filtered. The phosphorus content in the sample was determined via molecular absorption spectrophotometry (MAS; Hitachi; U 1100 Spectrophotometer, Tokyo, Japan) using the ascorbic acid method. In this method, in an acid medium, the ammonium molybdate and antimony potassium tartrate react with orthophosphate to form phosphomolybdic acid, which is reduced to intensely coloured molybdenum blue by ascorbic acid (SMEWW 4500-P E) [92]. The concentration of the remaining minerals in that set was determined via atomic absorption spectrometry (AAE) with an air-acetylene flame (Varian AA300, Mulgrave, VIC, Australia) (SMEWW 3111 B) [92]. The remaining elemental chemical characterisation of the ash was performed directly on the ash via scanning electron microscopy (SEM-EDS) using a Hitachi S4100 microscope equipped with a detector for energy-dispersive spectroscopy, with the elemental distribution being partially quantified using semiquantitative methods for spectrum analysis (EDS-EDAX with Bruker AXS detector; software: Quantax 200, Esprit version 1.9). 

### 3.7. Structural Characterisation of Pinus pinaster Cortex and Its Individual Fractions

*Fourier transform infrared spectroscopy (FTIR):* To analyse the chemical groups and bonding configurations present in the cortex of Pinus pinaster, as well as in its isolated fractions (cellulose, α-cellulose, holocellulose, and lignin), FTIR was employed. The FTIR analysis was conducted using a Thermo Scientific infrared spectrometer (Nicolet iS5 FTIR, Waltham, MA, USA) equipped with a diamond composite attenuated total reflectance (ATR) cell. The FTIR spectra were generated at a resolution of 4 cm^−1^, comprising a total of 32 scans and covering the frequency range from 4000 to 600 cm^−1^.

*Differential Scanning Calorimetry (DSC)*: The thermogravimetric analysis of the initial material (*Pinus pinaster* cortex) was conducted using a DSC (Perkin Elmer, Norwalk, CT, USA, DSC-7). The analysis was performed in the temperature range of 25 to 600 °C at a linear heating rate of 10 °C per minute.

*Scanning Electron Microscopy*: The micrographs of the initial material (*Pinus pinaster* cortex), along with its distinct fractions (cellulose, alpha-cellulose, holocellulose, and lignin), were captured utilising a field-emission scanning electron microscope (FEG-SEM) (Jeol JSM-7001F, Tokyo, Japan). The imagery was acquired employing a 5 kV voltage, at a 400 times magnification. To prevent electric charge buildup during observation, the samples intended for qualitative examination were precoated with an Au-Pd alloy layer.

*X-ray diffractometry*: A conventional X-ray diffractometer (Bruker D8 Advance DaVinci model, located in Karlsruhe, Germany) was employed for XRD analysis, using Cu-Kα radiation filtered through Ni (with a wavelength of λ = 0.15418 nm), generated at 40 kV and 30 mA. A linear detector (Lynxeye 1-D) was used to record the datasets in the range of 5° to 60° (2θ) with 0.02° (2θ) increments. Each data point was recorded at intervals of 0.5 s, and the rotation rate was maintained at 15 rotations per minute. The Rietveld refinement was carried out using Diffrac.eva v5.2 software, developed by Bruker AXS in Karlsruhe, Germany. In this process, the fundamental parameter approach was applied for phase quantification. The crystallinity index (CrI) was determined by calculating the intensities in the crystalline and amorphous regions following Equation (1) [83], representing the intensities in the crystalline and amorphous regions at peak located at approximately 2θ = 21.54–22.368° and 12.512–16.731°, respectively:

(1) $$CrI\;(\%)=\frac{I_{002}-I_{am}}{I_{002}}\times 100$$

where *I*_002_ = maximum intensity of the lattice diffraction, and *I_am_* represents the intensity of the amorphous diffraction.

## 4. Conclusions

The residues and by-products derived from pine are a significant reservoir of valuable biocompounds that hold substantial industrial value. Their extraction through the application of the biorefinery approach not only aids in their recuperation but also plays a pivotal role in advancing the principles of the circular economy. Total extractive materials with a higher value, approximately 10% *w*/*w* of the sample by mass, show potential use in a biorefinery. After lipophilic and hydrophilic extraction, the dry pine bark, approximately 90%, has high potential use as a raw material in industry and biorefineries. The findings indicate that the *Pinus pinaster* bark is primarily composed of lignin (51.55%) in terms of macromolecular compounds, followed by holocellulose (46.09%) and cellulose (23.21%). In the degradation stage, the *Pinus pinaster* bark exhibited a 10% reduction in mass when exposed to a temperature of 140 °C. Regarding the crystallinity index, both holocellulose and cellulose demonstrated comparable values, approximately around 25.5%. In contrast, α-cellulose displayed the highest crystallinity index, reaching 41%. This study shows that the low content of heavy metals in the pine bark may qualify this residue for various types of uses.

## Figures and Tables

**Table 1:** Chemical composition of dry pine bark from *P. pinaster* Aiton subsp. *atlantica*.

	Composition (% *w*/*w*)						
Parameter	This Study	Nunes et al. (1996) [31]	Simões et al. (2021) [10]	Ferreira-Santos et al. (2020) [32]	Fradinho et al. (2002) [27]	Santos et al. (2021) [28]	Vieito et al. (2019) [33]
Moisture	6.67 ± 0.01			8.15 ± 0.02			12.31 ± 0.20
Protein	1.94 ± 0.22			1.64 ± 0.03			12.82 ± 0.01
Total fat	0.98 ± 0.00			2.54 ± 0.26			1.60 ± 0.04
Ash	0.44 ± 0.01			0.87 ± 0.00		0.40 ± 0.02	1.75 ± 0.03
Holocellulose	46.09 ± 0.48				48.4 ^d^		
Cellulose	23.21 ± 0.57			17.39 ± 0.37 ^c^			
α-Cellulose	22.88 ± 0.02					23.2 ± 2.0	
Hemicellulose	23.21 ± 0.02 ^a^			12.31 ± 0.20		16.1 ± 3.8	
Lignins	51.15 ± 0.35	43.7 ± 2.4		41.65 ± 0.24			
Acid-soluble	2.85 ± 0.35			0.60 ± 0.00			
Klason	48.31 ± 0.31			41.05 ± 0.24	33.2 ^e^	46.2 ± 3.4	
Total extractives	9.75 ± 0.13	11.4 ± 2.2			16.6	11.97	
Toluene–ethanol (T:E)	5.61 ± 0.05						
Lipophilic (L)		2.3 ± 1.3	11.3 ± 1.7	2.54 ± 0.26	3.1	1.17 ± 0.09	
Ethanol (E)	1.54 ± 0.06	6.5 ± 1.2	19.5 ± 2.0	13.20 ± 0.31	10.3	8.71 ± 0.16	
(T:E) + (E) or (L) + (E)	7.15 ^b^	8.8	30.8	15.74	13.4	9.88	
Hydrophilic (H)	2.59 ± 0.10	2.6 ± 0.2	6.9 ± 1.7		3.2	2.09 ± 0.02	
%/[(L) + (E)] in total extractives	76.4	77.2	81.7		81.7	82.5	
[(L) + (E)]/(H)	2.9	3.4	4.7		4.5	4.2	

^a^ Hemicellulose (α-cellulose–holocellulose); ^b^ extractives: toluene–ethanol + ethanol; ^c^ estimated from the glucan content; ^d^ holocellulose determined after solvent extraction and alkaline extraction with 2% NaOH (0.5 h, 100 °C, 1 g/10 mL); ^e^ Klason lignin determined after solvent extraction and alkaline extraction with 2% NaOH (0.5 h, 100 °C, 1 g/10 mL).

**Table 2:** The CIELab system value of celluloses from *P. pinaster* Aiton subsp. *atlantica*.

Sample	Process.	L*	a*	b*	WI (%) ^1^
Crude cellulose	Hydrolysis with a mixture of acetic acid (CH_3_COOH) and nitric acid (HNO_3_)	81.21 ± 0.10	3.93 ± 0.12	19.27 ± 0.06	72.8
Holocellulose	Digestion with a mixture of acetic acid (C1H_3_COOH) and sodium chlorite (NaClO_2_)	79.92 ± 0.19	6.23 ± 0.20	20.11 ± 0.03	70.9
α-Cellulose	Hydrolysis with sodium hydroxide (NaHO)	82.71 ± 0.19	3.31 ± 0.20	11.13 ± 0.03	79.2

^1^ The whiteness index (WI%) was calculated using the equation $100-\sqrt{{\left(100-L^{*}\right)}^{2}+{a^{*}}^{2}+{b^{*}}^{2}}$; L* represents perceptual lightness; a* axis represents the red–green colour spectrum, with positive values indicating red and negative values indicating green; b* axis represents the yellow–blue colour spectrum, with positive values indicating yellow and negative values indicating blue.

**Table 3:** Fatty acid composition of maritime pine bark.

	This Study	Kurtça & Tumen (2020) [43]	Simões et al. (2021) [10]	Sousa et al. (2018) [42]
	(Relative Abundance %)			
Caproic acid (6:0)	-	-	-	0.403
Caprylic acid (8:0)	-	-	-	0.134
Pelargonic acid (9:0)	-	-	-	0.134
Lauric acid (12:0)	0.51 ± 0.003	-	-	-
Myristic acid (14:0)	1.10 ± 0.08	0.20 ± 0.20	-	0.134
Pentadecylic acid (15:0)	0.74 ± 0.02	-	-	0.134
Palmitic acid (16:0)	9.84 ± 0.38	6.50 ± 3.15	2.2 ± 1.7	7.38
Margaric acid (17:0)	0.54 ± 0.02	-	-	0.537
Stearic acid (18:0)	3.47 ± 0.24	0.45 ± 0.37	0.7 ± 0.3	3.09
Oleic acid (18:1*n*-9)	5.43 ± 0.24	13.91 ± 7.33	2.9 ± 1.0	6.44
Elaidic acid (18:1*n*-9)	-	-	-	0.671
Linoleic acid (18:2*n*-6)	2.35 ± 0.08	8.84 ± 4.72	-	2.95
α-Linolenic acid (18:3*n*-3)	-	0.37 ± 0.20	1.2 ± 0.0	-
Arachidic acid (20:0)	5.91 ± 0.22	1.92 ± 1.22	6.9 ± 1.8	8.99
Heneicosanoic acid (21:0)	0.71 ± 0.03	-	-	-
Behenic acid (22:0)	32.47 ± 0.21	3.10 ± 4.18	1.6 ± 0.3	33.7
Tricosylic acid (23:0)	2.45 ± 0.17	0.31 ± 0.06	0.8 ± 0.2	4.03
Lignoceric acid (24:0)	34.48 ± 0.28	3.19 ± 4.75	1.7 ± 0.5	28.6
Cerotic acid (26:0)	-	0.59 ± 0.76	-	2.68
Saturated fatty acid	92.22	20.41 ± 11.90	13.7 ± 1.1	89.9
Unsaturated fatty acid	7.78	23.31 ± 12.38	5.1 ± 1.0	10.1

**Table 4:** Mineral composition of maritime pine bark.

Element	Method	(mg/Kg Dry Matter)
Carbon (inorganic) (C)	SEM-EDS	199 ± 5.7
Oxygen (O)	SEM-EDS	2088 ± 16
Sodium (Na)	SEM-EDS	92.9 ± 1.4
Magnesium (Mg)	SEM-EDS	249 ± 1.2
Aluminium (Al)	SEM-EDS	456 ± 2.9
Silicon (Si)	SEM-EDS	125 ± 1.3
Phosphorus (P) ^1^	MAS-Vis	57.9 ± 0.63
Sulphur (S)	SEM-EDS	66.8 ± 0.51
Potassium (K)	SEM-EDS	134.2 ± 0.40
Calcium (Ca)	SEM-EDS	823 ± 13
Manganese (Mn)	SEM-EDS	34.1 ± 0.47
Iron (Fe)	SEM-EDS	65.4 ± 1.5
Cadmium (Cd)	AAS-Flame	<0.060
Lead (Pb)	AAS-Flame	<0.75
Copper (Cu)	AAS-Flame	1.76 ± 0.037
Chromium (Cr)	AAS-Flame	0.429 ± 0.0077
Nickel (Ni)	AAS-Flame	0.332 ± 0.017
Zinc (Zn)	AAS-Flame	3.11 ± 0.050
Total mineral content ^2^		4397 (0.44%)

^1^ SEM-EDS result: 61.8 ± 1.7 mg P/kg dry matter; ^2^ ∑ mineral concentrations; minerals: C, O, Na, Mg, Al, Si, P, S, K, Ca, Mn, Fe, Cu, Cr, Ni, and Zn.

## Data Availability

Data are contained within the article.
